# Knowledge, competence, experience of healthcare practitioners using glucometers for patient care in Nigeria

**DOI:** 10.4102/ajlm.v14i1.2770

**Published:** 2025-10-07

**Authors:** Salisu B. Muazu, Hauwa Bako, Ahmad M. Bello, John N. Onuche, Faruk Salami, Abimbola O. Abioye, Zainab I. Nadabo, Richard J. Banya, Eni-yimini S. Agoro, Saheed A. Adekola

**Affiliations:** 1 Department of Internal Medicine, Faculty of Medicine, Rasheed Shekoni Federal University Teaching Hospital, Dutse, Nigeria; 2 Department of Medical Laboratory Science, Faculty of Allied Health Sciences, Ahmadu Bello University Zaria, Zaria, Nigeria; 3 Department of Medical Laboratory Science, Faculty of Allied Health Sciences, Bayero University Kano, Kano, Nigeria; 4 Kogi State Hospital Management Board, Lokoja, Nigeria; 5 Laboratory Department, Okene Zonal Hospital, Okene, Nigeria; 6 Department of Clinical Pathology, Faculty of Medicine, Lagos University Teaching Hospital, Lagos, Nigeria; 7 Laboratory Department, Women and Children Hospital, Gombe, Nigeria; 8 Faculty of Medical Laboratory Science, Taraba State College of Health Technology, Takum, Nigeria; 9 Department of Biochemistry, Faculty of Basic Medical Sciences, Federal University Otuoke, Bayelsa, Nigeria; 10 Department of Medical Laboratory Science, Faculty of Clinical Pathology, Federal Medical Centre, Lagos, Nigeria

**Keywords:** knowledge, competence, experience, glucometers, healthcare practitioners, Nigeria

## Abstract

**Background:**

Despite the widespread acceptability of glucometers as a blood glucose self-monitoring and point-of-care device, their usage is confronted with operational, technical, regulatory, and quality control concerns.

**Objective:**

This study assessed knowledge, estimated competence, and measured experience of healthcare practitioners using glucometers for patient care in two states of Northern Nigeria.

**Methods:**

This cross-sectional, descriptive study used a total population sampling strategy and self-completed questionnaires. A total of 768 questionnaires were distributed to hospitals in Jigawa and Kogi States, Nigeria, from December 2019 to April 2022. The questionnaire had three sections: collecting details about type of healthcare facility, sociodemographic characteristics and educational qualifications of participants, and assessment of knowledge, competence and practice. Data were analysed and results expressed as frequencies and percentages.

**Results:**

Overall, 570 questionnaires were filled and retrieved, giving a response rate of 74.2%. Most of the participants were male (312; 54.7%); female participants totalled 258 (45.3%). Most participants were aged < 50 years (25–40 years, 215 [37.7%]; 41–50 years, 246 [43.2%]). The majority of participants were Medical Laboratory Scientists (124, 21.8%]), Technicians (151, 26.5%), or Nurses (132, 23.2%). Most participants (284, 49.8%) reported having no formal training prior to first use of glucometers in patient care. Many participants (379, 66.5%) knew about glucometer calibrators; 235 (41.2%) did not know what specific purpose calibrators served.

**Conclusion:**

This study found a lack of knowledge, competence and experience among healthcare practitioners, especially for hands-on use of glucometer calibrators and standard operating procedures for blood glucose testing using glucometers.

**What this study adds:**

The study brings to fore the need for training and retraining of healthcare practitioners on the theoretical and practical skills required for operating glucometers. Periodic calibration of glucometers and provision of quality control materials should be incorporated into standard operating procedures at point-of-care testing workstations in health facilities.

## Introduction

The practice of using glucometers for self-monitoring of blood glucose (SMBG) is an essential component for care and management of diabetes,^1,2^ which is supported by educational and clinical guidelines.^3,4^ Information generated from SMBG is shared with healthcare teams for modification of multiple self-care behaviours and simplifying problem-solving discussions.^5,6^ Numerous sources of error have been reported to affect blood glucose testing using glucometers, including clinical, operators, reagent, environmental, software, and hardware.^7^ Despite the myriad of challenges, glucometers have undergone evident technological and methodological improvements in recent times.^8^ Although conflicting reports exist pertaining to the accuracy and reproducibility of glucometers, evaluation have showed decreased reliability and non-compliance with some international standards of certain brands,^8,9^ while increased sensitivity and specificity have been reported for other brands.^10,11^ Compared with central laboratory testing for blood glucose using auto-analysers, enhanced performance has been observed for most glucometers, despite quality control concerns.^9,12,13^

Outpatient glucometers differ from those found in hospital settings, resulting in heterogeneity of operators and multi-directional studies. Because of the diversity of glucometer operators, some studies^14,15,16,17,18,19^ have assessed the knowledge, attitude and practice of individuals living with diabetes type 1 and 2 using glucometers for routine SMBG, while other studies^20,21,22,23,24,25^ evaluated knowledge, competency and experience of healthcare practitioners (HCPs) operating glucometers.

Researchers’ efforts to develop an operator’s assessment checklist^26^ and a self-management model^27^ for HCPs and patients are yet to be fully recognised and accepted by international regulatory organisations. Ensuring patient safety and reducing medical errors are key components of global best practices.^28^ Integrating point-of-care tests (POCTs) into laboratory management information systems is necessary in order to allow bidirectional information exchange and traceability.^29^ The lack of or inadequate HCP formal training, as well as the absence of or inadequate quality control materials and practices, have been reported as major concerns for using glucometers for blood glucose monitoring and patient care.^30,31,32,33^

Studies conducted in Northern Nigeria have shown poor quality control practices for glucometers, and inadequate or lack of training for HCPs, to be major challenges facing the use of glucometers as a point-of-care device in the region. Although studies have accessed either knowledge, competence or experience among HCPs, limited studies have investigated the trio in Jigawa and Kogi States of Nigeria, where the study was conducted. The objectives of the study were to assess knowledge, estimate competence and measure experience of HCPs using glucometers for patient care in two states of Northern Nigeria.

## Methods

### Ethical considerations

An application for full ethical approval was made to the Jigawa State Health Research Ethics Committee and Kogi State Health Research Ethics Committee. Ethical approval reference number MOH/SEC/3/784/1/12 received on 23 November 2019 and reference number MOH/PRS/465/V.1/011 received on 17 May 2021, were granted before the study commenced in Kogi and Jigawa States. We obtained written informed consent from all participants. Information which revealed the identity of participants was masked for confidentiality purposes. Study materials were stored in locked steel cabinets accessible only to the research team.

### Study design and setting

We performed a cross-sectional descriptive assessment conducted using a self-completed questionnaire (Online Supplementary Questionnaire). The questionnaire was designed to cover important aspects of knowledge, competence and experience required by health professionals to appropriately use glucometers for patient care.

The study was performed in 36 health facilities of Kogi and Jigawa States of Nigeria. Healthcare practitioners comprising physicians, medical laboratory scientists, pharmacists, physiotherapists, nutritionists and/or dietitians, medical laboratory technicians, community health workers, and nurses, spanning 48 Local Government Areas, participated in the study between 02 December 2019 and 16 October 2020 in Jigawa State, and from 18 May 2021 to 29 April 2022 in Kogi State.

### Sampling size and strategy

Using the total population sampling approach, all eligible HCPs working in the study hospitals and involved in patient care, who served as the target population, were invited to take part in the study. An equal number of questionnaires was distributed to the respective departments in the study hospitals.

Healthcare practitioners who were on intern service or the mandatory national youth service corps were excluded. Health professionals in administrative positions were also excluded, because they were not actively involved in direct patient care.

### Data collection

A questionnaire for self-completion was developed by the investigators through an iterative process involving several stages of drafting and revision of the questionnaire items. The drafting and redrafting process involved experts in different fields with expertise in survey research, laboratory quality management systems, and POCT to ensure the questionnaire’s content and face validity.

The questionnaire was pre-tested with a small group of health professionals (*n* = 30) from 25 November 2019 to 29 November 2019 at the accident and emergency ward of Rasheed Shekoni Federal University Teaching Hospital, Jigawa State, and the instrument was refined using the outcome of aggregate score for sections, as well as clarity and interpretation of the questions. Participants of the pilot study were asked to make inputs to improve the questionnaire, and minimal adjustments which did not show need for significant changes were suggested. As a result, the questionnaire was adopted for the study.

The questionnaire was designed to evaluate the knowledge, competence, and experience of HCPs on the use of glucometers in patient-care settings. Questions were numbered serially, did not follow a particular design, and were attached to options and sub-questions. The final questionnaire was structured into three sections; section ‘A’ comprised facility details, section ‘B’ contained personnel details (sociodemographic characteristics of respondents), and section ‘C’ comprised assessment questions for knowledge, competence and experience. Questions in section ‘C’ included general knowledge about glucometers and calibrators, technical competence, and experiences of HCPs actively operating glucometers in patient care.

A total of 768 copies of the final paper-based questionnaires were distributed to participants at their various workstations. Reminder/follow-up was done via phone calls at weekly intervals for three consecutive weeks, after which manual collection of paper-based completed questionnaires was performed.

### Data analysis

Information from completed questionnaires was entered into a Microsoft® Excel database, (Microsoft Corporation, Redmond, Washington, United States). Data entry was performed by two research assistants. Entry by the first research assistant was cross-checked and validated by the second. Missing data were not encountered during data entry by the research assistants and review by the research team. Data entered were cleaned then exported to Statistical Package for the Social Sciences v25.0 (IBM Corp., Armonk, New York, United States) for subsequent management and analysis. Gender, age range and educational qualifications were represented by a specific code prior to data entry for categorisation purposes. Respondents’ characteristics are expressed in percentages and frequencies analysed using simple descriptive statistical tools.

Each knowledge question was scored as 0 for incorrect responses and 1 for correct responses. Composite knowledge was calculated as an aggregate of the six knowledge questions, with a minimum of 0 and a maximum of 6 achievable by each respondent. Respondents who achieved scores between 0 and 4 (< 70%) were considered to have low knowledge, while those who scored 5 and 6 (≥ 70%) were considered to have high knowledge. Similarly, competence was operationalised by a mix of 6 questions which were scored and analysed in the same way as described for the knowledge domain.

Categorical variables were analysed using a 2 × 2 cross-tabulation analysis (Table 1) to determine the level of knowledge and competence of respondents, hypothesised as a function effect of formal training and availability of standard operating procedures (SOPs) for glucometers. Experience was measured by the number of positive responses related to skills and work experience pertaining to glucometer usage.

**TABLE 1:** Cross-tabulation (2 × 2) of responses collected from 02 December 2019 to 16 October 2020 in Jigawa State, Nigeria, and from 18 May 2021 to 29 April 2022 in Jigawa and Kogi States, Nigeria.

Respondents’ knowledge and competence	Formal training (≥ 70%)		No formal training (< 70%)	
	*n*	%	*n*	%
**Formal training**				
High knowledge	217	38.0	125	22.0
Low knowledge	74	13.0	154	27.0
High competence	171	30.0	17	3.0
Low competence	114	20.0	268	47.0
**Availability of standard operating procedures**				
High knowledge	165	29.0	167	29.3
Low knowledge	83	14.6	155	27.1
High competence	123	21.6	69	11.9
Low competence	125	21.9	253	44.6

## Results

### Participant characteristics

Of the 768 questionnaires distributed, 570 were filled and retrieved, with a response rate of 74.2% recorded. The majority of the respondents were Medical Laboratory Technicians (*n* = 151, 26.5%), Nurses (*n* = 132, 23.2%), or Medical Laboratory Scientists (*n* = 124, 21.8%) (Table 2). The highest educational qualification of respondents was almost evenly split between the diploma level (*n* = 260, 45.6%) and graduate degree level (*n* = 245, 43.0%).

**TABLE 2:** Participants’ sociodemographic characteristics, collected from 02 December 2019 to 16 October 2020 in Jigawa State, Nigeria, and from 18 May 2021 to 29 April 2022 in Jigawa and Kogi States, Nigeria.

Characteristic	*n*	%
**Gender**		
Female	258	45.3
Male	312	54.7
**Age (years)**		
25–40	215	37.7
41–50	246	43.2
51–60	101	17.7
> 61	8	1.4
**Educational qualification**		
Certificate	23	4.0
Diploma	260	45.6
Graduate degree	245	43.0
Postgraduate	42	7.4
**Professional cadre**		
Physician	69	12.1
Medical Laboratory Scientist	124	21.8
Pharmacist	25	4.4
Nurse	132	23.2
Medical Laboratory Technician	151	26.5
Pharmacy Technician	15	2.6
Community Health Extension Worker	51	8.9
Nutritionist/Dietitian	3	0.5
**Professional experience (years)**		
1–10	235	41.2
11–20	219	38.4
21–30	87	15.3
> 30	29	5.1

### Knowledge and competence of respondents

Most respondents (379/570, 66.5%) were familiar with glucometers as a point-of-care device (Table 3). Despite availability of training for HCPs in some hospitals (248/570, 43.5%), most HCPs have no theoretical (332/570, 58.2%) and practical skills (368/570, 64.6%) on glucometer calibrators.

**TABLE 3:** General responses of healthcare professionals, collected from 02 December 2019 to 16 October 2020 in Jigawa State, Nigeria, and from 18 May 2021 to 29 April 2022 in Jigawa and Kogi States, Nigeria.

Questions	Responses	
	*n*	%
**Knowledge of existence of glucometers**		
Yes	379	66.5
No	191	33.5
**Knowledge of specific use of glucometers**		
Blood glucose monitoring	558	97.9
I don’t know	12	2.1
**Practical use of glucometers in patient care**		
Yes	499	87.5
No	71	12.5
**Formal training on glucometers**		
Yes	286	50.2
No	284	49.8
**Knowledge of glucometer calibrators existence**		
Yes	379	66.5
No	191	33.5
**Knowledge of specific use of glucometer calibrators**		
Blood glucose testing	118	20.7
Checking precision of device	335	58.8
I don’t know	117	20.5
**Theoretical knowledge of how to calibrate glucometers**		
Yes	238	41.8
No	332	58.2
**Skills for calibration of glucometers**		
Yes	202	35.4
No	368	64.6
**Frequency of glucometer calibration**		
Daily	73	12.8
Weekly	91	16.0
Monthly	91	16.0
Annually	40	7.0
Never	275	48.2
**Inconsistency of results obtained from same glucometer in use**		
Yes	285	50.0
No	273	47.9
Not Sure	12	2.1
**Rating of accuracy of glucometers compared to central laboratory testing (%)**		
0–10	31	5.4
11–20	25	4.4
21–30	35	6.2
31–40	33	5.8
41–50	97	17.0
51–99	249	43.6
100	100	17.6
**Availability of training on glucometers in hospitals**		
Yes	428	75.1
No	141	24.7
I don’t know	1	0.2
**Availability of Standard Operating Procedures for glucometers in hospitals**		
Present	248	43.5
Absent	322	56.5
**Observation of variations in test results compared to central laboratory testing**		
Yes	310	54.4
No	259	45.4
Not Sure	1	0.2
**Observation of discordant results from same patient using the same device**		
Often	79	13.9
Quite Often	118	20.7
Not Often	238	41.8
Never	120	21.1
Not Sure	15	2.7

Many (368/570, 64.6%) had never calibrated a glucometer used for patient care. Standard operating procedures for blood glucose testing using glucometers were unavailable in most clinical settings (322/570, 56.5%).

## Discussion

Our study found a paucity of theory and skills for glucometer calibrators and calibration in about half of the participants, which in turn impacts the frequency of calibration of the device by HCPs, reliability and reproducibility of test results obtained, and the quality of life of the patients. Unavailability of SOPs for blood glucose testing using glucometers was reported by many respondents, despite the accessibility of training in some healthcare facilities.

About two-thirds of participants knew glucometers existed as a point-of-care device utilised for patient care. This is because over the years, glucometers have gained wide acceptability as an SMBG tool and point-of-care device. A study^23^ at Tygerberg Hospital located in the northern suburbs of Cape Town, Western Cape, South Africa, in 2016, which assessed knowledge and awareness of POCTs, reported that 97% of participants had performed blood glucose testing using glucometers. However, by restricting participation to only doctors and nurses, the study excluded other HCPs who were likely to operate the device. Another study^22^ in Nigeria, at the University of Benin Teaching Hospital and Central Hospital, Edo State, Southern Nigeria, in 2015, reported good knowledge of POCT (50.6%) among doctors, but the finding is not comparable to our study because of non-specification of POCT devices and restricted enrolment of study participants to only doctors.

We observed that work experience, or years of service, for most respondents ranged between 1 and 10 years (41.2%) and 11 and 20 years (38.4%), showing that years of HCPs’ service or work experience in patient care was not directly linked with knowledge about existence of the device and its use. This implies that HCPs may not have to practise for many years before becoming acquainted with glucometers. Similarly, an assessment of nurses’ competencies in diabetes care,^20^ in Anambra State, Southeast Nigeria, in 2023, showed a positive association between duration of work experience and skills. However, the skills were diverse and not limited to blood glucose testing using glucometers. As a result, deductions from the study aren’t comparable to our study.

Nearly half of the respondents (49.2%) reported not securing formal training or certification prior to first use of glucometers in patient care. This is because most health facilities in the private or public sector in Nigeria rely solely on device manual inserts provided by manufacturers to guide HCPs as a substitute for formal training or certification. Similarly, a multi-centre study by Nnakenyi et al.,^30^ conducted in Enugu Southeast Nigeria in 2017 across five health facilities, found documentation for personnel training in only 26% of POCT sites, for which glucometers accounted for about 65% of the point-of-care devices in use. Inadequate formal training^22,23^ for HCPs remains a major setback for achieving accuracy and reliability in blood glucose testing using glucometers. Lack of or inadequate training translates to limited theoretical knowledge^20^ and skills that may negatively impact quality assurance, quality control, and quality management systems, as a whole. Limited knowledge of operators, particularly HCPs, can lead to misdiagnosis, inaccurate test results, flawed research reports and wrong therapeutic interventions. When knowledge is poor, practice is hampered, hence the need to educate HCPs on the importance of seeking knowledge and skills required for operating point-of-care devices such as glucometers.

Some studies have suggested in-service training on POCTs for doctors, nurses and midwives^20,21,22,23^ in Nigeria and South Africa. The need to train other cadres of HCPs is less advocated in studies, despite the presence of such professionals in a standard POCT organogram as stipulated in international guidelines. Three-quarters of participants (75.1%) reported in the affirmative regarding availability of a training forum for HCPs on glucometers in their health facilities. Despite the availability of the training forums, HCPs do not deem it necessary to secure formal training or certification preceding first use in patient care. Hospital in-service training provides an avenue for HCP continuous professional education and development. Other sources, such as conferences, journal articles, and pharmaceutical companies, have also served as sources of information for HCPs on POCT.^31^

Although more than half of respondents (66.5%) were aware of the existence of glucometer calibrators, many (41.2%) didn’t know what purpose calibrators specifically served in SMBG. This may be attributed to the fact that most new glucometers do not come with calibrators in their packs, and those with calibrators lacked an operation manual.^13^ Thus, the necessity to harmonise theorical knowledge and skills for most HCPs. More than half of respondents (58.2%) did not possess theoretical knowledge about glucometer calibration, and a similar number of respondents (64.6%) had no practical experience of glucometer calibration, which is supported by reports of some studies^11,23^ conducted in South Africa, 2020, and Australia, 2025. Theoretical knowledge is crucial for operators of glucometers in order to identify factors which may either affect the testing device or strips, leading to underestimation or overestimation of blood glucose when using the device.

The essence of calibration is for early detection of device non-conformances and ensuring results obtained are accurate and reproducible. Studies in Douala, Southwest Cameroon,^8^ in 2014–2015 and Calabar, Southsouth Nigeria,^9,13^ in 2021, have evaluated the functional capacity of glucometers using calibrators provided by some brands. The frequency of glucometer calibration varied among respondents and about half (48.2%) reported to have never calibrated a glucometer, despite years of service in patient care. Responses showed that the ideal way of calibrating glucometers which is before first use, weekly, and after purchasing a new vial of test strips is not practised by most HCPs in Nigeria. Likewise, Inaku et al.,^32^ in a survey of POCT use among doctors and nurses in Calabar, Southsouth Nigeria, 2019, reported that 24.3% of participants performed quality control practices on POCTs in use of which glucometers accounted for 91.4% of the POCTs. The study reported poor quality control among doctors and nurses operating the devices. Regulation of POCT use was also suggested by the majority of respondents in the study. Limited knowledge of glucometer calibration among HCPs,^9^ as reported in a study in Calabar, Southsouth Nigeria, 2019, translates to erroneous results and unsatisfactory patient outcomes,^13^ as supported by a study in Enugu Southeast Nigeria, 2022. Periodic calibration of glucometers is recommended in order to prevent misdiagnosis, improve test result quality, minimise mismanagement of patients and achieve beneficial outcomes that will impact quality of life of end-users.

Certain brands of glucometers have exhibited superiority in terms of precision and accuracy, even without necessarily meeting certain international standards for POCTs.^8,10,11^ This has led to product bias among HCPs and patients, with increased competition for manufacturing companies. Healthcare practitioners are expected to know when results tend towards incorrectness, especially when managing high-risk patients such as those on admission in intensive care units, to prevent misdiagnosis.^23^ Half of the participants (50.0%) reported observing inconsistency of test results obtained from the same glucometer operated at different times. The finding may be attributable to the myriad of technical challenges and sources of errors associated with blood glucose testing using glucometers.

About half of respondents (43.6%) rated the accuracy of glucometers as 51.0% to 99.0%, and 17.6% reported its accuracy as 100.0% when compared to central laboratory testing for blood glucose. In recent times, increased accuracy and precision for some glucometers have been reported,^2,10^ although there are contrary findings.^8,9^ The comparison of our finding with other reports may not be accurate because of differences in study designs: descriptive cross-sectional versus comparative analytical prospective. Glucometer operators should be knowledgeable about operational limitations of certain brands of the device used for blood glucose testing. Suggestions to keep point-of-care devices, such as glucometers, under the laboratory department has been faced with a lot of opposition, because such devices are dedicated for near patient testing. Healthcare practitioners should be equipped with knowledge of limitations of the device and when to refer testing for critical patients to the central laboratory.

Standard operating procedures are sets of step-wise instructions containing elements of quality assurance and quality control which conform with regulatory guidelines for end users. Robust training, supervision and intensive quality assurance mechanisms are necessary to optimise blood glucose testing using glucometers.^34^ Unavailability of SOPs for glucometers in most health facilities accounted for 56.5% of responses. Provision of teaching aids and SOPs in the form of flow charts or simple checklists for glucometers is essential in achieving quality blood glucose testing. Absence of SOPs for blood glucose testing using glucometers in health facilities may impact negatively on operators’ proficiency and quality of results generated using the device.^23^

Most studies in sub-Saharan Africa^22,23,30^ have broadly assessed POCTs as an entity and considered glucometers only as a subset of POCT, with study participation limited to specific healthcare cadres: doctors, nurses, midwives, or laboratory professionals. For instance, an multi-centre study^30^ in Enugu Southeast Nigeria, 2017, glucometers accounted for 65% of the POCTs, and participation was restricted to doctors, nurses, and laboratory personnel. The notable findings were lack of traceable patient test records, troubleshooting faulty devices by non-laboratorians, and poor method validation observed at the study sites. Absence of a POCT committee in all study hospitals was observed in all study sites. Another study^22^ in Edo State, Southern Nigeria, in 2015, conducted at two tertiary health facilities assessed knowledge and utilisation of POCT among doctors. The study did not specify the types of POCTs assessed, what was assessed, the reason for the assessment, and participation was restricted to only doctors. A hospital audit^23^ at Cape Town, Western Cape, South Africa, in 2016, which evaluated the knowledge of POCT best practices among clinical staff, precisely doctors and nurses, suggested the need for formal training and competency assessment for operators. The reasons for limiting participation to doctors and nurses only was not clearly defined. A survey of POCT devices among doctors and nurses^32^ at Calabar, Southsouth Nigeria, in 2019, also showed that most operators used POCT devices for disease monitoring. The POCTs evaluated were glucometers, urine testing strips, haemoglobin meters, bilirubin meters, and cholesterol meters. The non-restriction of the POCTs to glucometers, differences in study designs, and limitation of participation to specific cadres of HCPs in other studies, may not allow for appropriate comparison with the present study.

In Nigeria, operating glucometers in hospital settings is not restricted to a specific cadre of HCPs.^22^ All HCPs involved in patient care, irrespective of training, years of experience and skills, automatically become operators of the device^32^ because of the lack of organograms for POCTs in Nigerian hospitals. The unavailability of hospital-based POCT committees may also be responsible for some irregularities observed in some health institutions. Our study was carefully designed to identify gaps and proffer solutions to the spate of blood glucose testing using glucometers in Nigeria.

Currently, there is no regulatory agency or national guideline in Nigeria dedicated to POCTs or glucometers, and there are no POCT committees in hospitals. Organising centralised local, state or national POCT training or certification in Nigeria may encourage stakeholders in government, health, science, and technology, as well as the private sector, towards engagement of HCPs in developing policies, creating hospital committees on POCTs, and implementation of policies at the grassroots.

### Recommendations

We recommend compulsory formal training, on-the-job competency assessment, and certification on glucometers and other POCTs for HCPs. Updating and assessing HCPs’ knowledge, competency and skills is a professional gap that needs to be addressed by the relevant stakeholders. Formation of hospital-based, state and national POCT committees, provision of standard protocols, and quality control materials and engagement of central laboratories in establishing standard POCT guidelines and implementation of its content should also be considered.

### Limitations

Our study limitations include the restriction of participation to HCPs employed only by the study hospitals which are directly under the state ministries of health, thereby excluding HCPs employed in the private sector although actively involved in patient care. Although the response rate was quite high, we observed in some hospitals that the available HCPs were busy with official duties when the questionnaires were distributed and didn’t have the time to fill the questionnaires at follow-up and until completion of the study. The questionnaire was not designed to get feedback from HCPs on how POCT committees will be formed at hospitals, the likely members of the proposed committees, and the number of POCTs to be included in local and national guidelines. The information would have provided insights into the opinions of HCPs on POCTs, and provided suggestions on how the state and federal ministries of health can establish a formidable POCT programme.

Our findings are solely applicable to the study hospitals and may not be generalised for all health facilities in Nigeria, especially in resource-constrained settings where testing of blood glucose is completely absent or only performed in central laboratories, as seen in some hospitals.

### Conclusion

The study found that although most participants possessed theoretical knowledge, the skills required for calibration of glucometers were absent. Standard operating procedures, calibrators, formal training, and skills acquisition forums for glucometers should be made available in health facilities in order to improve test quality and minimise avoidable errors in blood glucose testing using glucometers in Nigeria. Our study is the first in Nigeria conducted among a diverse cadre of HCPs, numerous inter-state health facilities, and different tiers of health centres: cottage, primary, secondary, and tertiary hospitals.
